# Fetal and Maternal Outcomes in a Cohort of Patients With Primary Sjogren’s Syndrome: An Observational Study

**DOI:** 10.7759/cureus.66926

**Published:** 2024-08-15

**Authors:** Vinya Paladugu, Nikhil Teja, Rajashree Menon, Riju Ramachandran

**Affiliations:** 1 Obstetrics and Gynaecology, Amrita Institute of Medical Sciences and Hospital, Kochi, IND; 2 Internal Medicine, Amrita Institute of Medical Sciences and Hospital, Kochi, IND; 3 General Surgery, Amrita Institute of Medical Sciences and Hospital, Kochi, IND

**Keywords:** congenital heart block, sjogren’s syndrome, autoimmune disease, fetal outcomes, maternal outcomes, pregnancy

## Abstract

Background

Pregnant women with primary Sjogren’s syndrome (PSS) have a high incidence of maternal and fetal complications due to immunological variations caused by maternal antibodies (anti-Sjogren's-syndrome-related antigen A (SSA) and anti-anti-Sjogren's-syndrome-related antigen B (SSB) crossing the placenta from the 12th week of gestation, mediating the tissue damage. A multidisciplinary approach is required in the management of such patients. Data regarding the effects of PSS on pregnancy are deficient in the Indian context.

Methods

This was a retrospective observational study on the maternal and fetal outcomes of PSS on a cohort of pregnant women treated at our tertiary care center between 2011 and 2020. Patients who satisfied the criteria for PSS were included, and patients with other associated autoimmune disorders were excluded. Maternal age, number of miscarriages, prior obstetric history, and maternal and fetal complications were recorded and statistically analyzed.

Results

There were 16 pregnancies in 10 women with PSS (incidence: 1/1,000 pregnancies/year) in our study. The mean gestational age of the mother at presentation was 31 ± 9.0 weeks. Oligohydramnios in five (11.8), intrauterine fetal demise (IUFD) in two (11.8), and first-trimester medical termination of pregnancy (MTP) in four (23.5) were noted. The weight of neonates was 2.3 ± 0.8 kg, and the mean duration of neonatal intensive care (NICU) stay was seven days. Fetal echo revealed congenital heart block (CHB), with six (50.0%) complete and one (8.3%) incomplete (p = 0.004). One baby needed a permanent pacemaker.

Conclusion

Maternal and fetal complications are high in our set of mothers with PSS. Early detection, regular follow-up, and a multidisciplinary approach may improve the outcome.

## Introduction

Autoimmune disease (AD) results when the body produces antibodies against itself. In most instances, the events that initiate this response to self-molecules are unknown. Several studies associate environmental, genetic, and certain types of infections with the initiation of autoimmunity [1]. AD may be organ-specific or systemic. Organ-specific AD commonly affects the thyroid (Hashimoto's thyroiditis, thyrotoxicosis), stomach (pernicious anemia, autoimmune, atrophic gastritis), adrenal glands (Addison’s disease), and pancreas (type of insulin-dependent diabetes mellitus (IDDM)) [2]. The systemic variants of AD are rheumatoid arthritis (RA), systemic lupus erythematosus (SLE), scleroderma, and rare disorders like Sjogren's syndrome (SSJ) and other vasculitis disorders. An increased risk of miscarriage and reduced fecundity causes concern in reproductive women having an AD. The changes associated with pregnancy produce immunological variations in patients with AD. Hence, these disorders play a crucial role during pregnancy and should be dealt with with the utmost care. Commonly encountered AD during pregnancy are SLE, RA, and anti-phospholipid antibody syndrome. SSJ accounts for a fraction of these [3].

SSJ is an AD due to chronic inflammation very commonly affecting the exocrine glands. Primary Sjogren's syndrome (PSS) is the term used to describe the disorder when it presents itself. Secondary Sjogren’s syndrome (SSS) is the term used to describe SSJ that is linked to additional underlying connective diseases (CDs), such as RA and SLE. SSJ impacts women up to nine more often than men, with an incidence ranging from 0.1% to 4.8% [4]. Rheumatologists frequently utilize the American College of Rheumatology/European League Against Rheumatism (ACR-EULAR) classification criteria for PSS to classify it as PSS [5]. Clinical symptoms of SSJ can vary widely, ranging from mucosal surface dryness to systemic involvement or extra glandular signs. The defining feature of the disease is the exocrine glands' localized lymphocytic infiltration. SSJ is confirmed in a lab setting by identifying antinuclear antibodies, notably anti-Sjogren's-syndrome-related antigen A (SSA) (anti-Ro), anti-Sjogren's-syndrome-related antigen B (SSB) (anti-La), cryoglobulins, and hypocomplementaemia. These are the primary prognostic markers of SSJ [6,7]. Pregnant women with SSJ are likely to experience more maternal and fetal complications. These antibodies (anti-SSA and anti-SSB) cross the placenta, usually from the 12th week of gestation, mediate the tissue damage and lead to complications [7-12]. Hence, the knowledge of obstetric changes and careful monitoring of patients with connective tissue disorders, especially PSS, helps in the early detection and treatment of adverse events. The management of patients with PSS requires a multidisciplinary regimen where obstetricians, neonatologists, radiologists, rheumatologists, and pediatric cardiologists work together for safer maternal and fetal outcomes.

Numerous studies have shown the highly variable rate of fetal and maternal risks in patients with SSJ. Most research indicates that this disease is associated with a higher risk of preterm deliveries, preterm premature rupture of membranes (PPROM), cesarean sections, catastrophic fetal outcomes in pregnancies, and lower mean neonatal birth weight in the children. A pathologic intrauterine fetal growth restriction that is independent of the timing of delivery has been proposed as the cause of this [7,12]. Two concerning fetal consequences of PSS include congenital heart block (CHB) and neonatal lupus [13]. The more serious fetal problem, CHB, results from antibodies against either anti-SSA, anti-SSB, or both, damaging the atrioventricular node. According to Western research [14], the risk of CHB in an anti-SSA/Ro-positive pregnancy is 1-2%.

Considerable studies have been done on the impact of several autoimmune disorders, such as SLE and antiphospholipid antibodies, on the outcome of pregnancy. Unfortunately, there is a paucity of studies in SSJ about the effects of pregnancy on the Indian population. Our aim is to look into the effects of PSS on pregnancy from an Indian perspective, both for the mother and the fetus.

## Materials and methods

This was a retrospective observational study on a cohort of pregnant women with PSS who were treated at our tertiary care center between 2011 and 2020. The obstetric department at our institution manages 1,200-1,500 pregnancies annually. Prior to starting the study, clearance was obtained from the institutional review board and the ethics committee (ECASM-AIMS-2021-349). The study was conducted pursuant to the 1964 Helsinki Declaration's guidelines for ethics. The study was then registered with the clinical trial registry (CTRI/2022/04/041676) before retrieving data from the hospital records.

Data were retrieved from the hospital electronic database using the parameters "pregnancy," "Sjogren's," "systemic lupus erythematosus," "rheumatoid arthritis," "fetus with congenital heart block," and "neonatal lupus." Patients diagnosed by their treating rheumatologist as PSS and presenting to our department with pregnancy were included, and patients with other associated CD were excluded.

The patients' demographic details, such as their age, the number of miscarriages they had, and their previous obstetric history, were noted. Preterm delivery (delivery before 37 weeks of gestation), intrauterine growth restriction (IUGR) with or without Doppler changes, and oligohydramnios/polyhydramnios (minimum vertical pocket 2 cm, maximum vertical pocket >8 cm, respectively) were some of the pregnancy parameters that were recorded. Birthweight less than 10th centile for gestational age, or small for gestational age (SGA), as well as medical termination of pregnancy (MTP) or intrauterine fetal demise (IUFD), were noted.

The type of AD, prenatal drugs, fetal Doppler, fetal echocardiogram, CHB, and neonatal lupus were the parameters noted. Additionally, the umbilical artery pH, birthweight, gestational age, delivery method, Apgar's score, and babies with CHB were noted. Follow-up information on the postnatal health status of the affected women and their babies was enquired telephonically.

All data were compiled on a Microsoft Excel sheet (Microsoft Corp., Redmond, VA) and statistically analyzed using SPSS (IBM SPSS Statistics for Windows, Version 22.0., IBM Corp., Armonk, NY). Categorical variables are expressed using frequency and percentage. Numerical variables are presented using mean and standard deviation. The chi-square test was used to find the association of PSS and SLE with the outcome variables. When a p-value was less than 0.05, it was deemed statistically significant. The manuscript was prepared in alignment with the Strengthening the Reporting of Observational Studies in Epidemiology (STROBE) criteria checklist for cohort studies.

## Results

There were 97 pregnancies complicated by autoimmune disorders encountered in our obstetric department out of 14,400 pregnancies between January 2010 and December 2019. There were 42 pregnancies satisfying the search parameters and having SSA and SSB positivity. Since the aim of our study was the assessment of pregnant women with PSS, we excluded 26 pregnancies having associated SLE (n = 15), RA (n = 2), and other mixed connective tissue disorders (n = 9). We had 16 pregnancies in 10 women with PSS in our study (incidence: ~1/1,000 pregnancies per year).

The mean gestational age of the pregnancy with PSS at presentation was 31 ± 9.0 weeks. There were oligohydramnios in five (11.8%), IUFD in two (11.8%), and first-trimester MTP in four (23.5%) pregnancies. We had eight (80%) term deliveries and two (20%) preterm deliveries, including one (5.9%%) preterm vaginal delivery. IUGR was seen in two (11.8) pregnancies.

Hydroxychloroquine (n = 10) (62.5%), steroids (n = 5) (31.25%), and azathioprine (n = 4) (25%) were the drugs taken by the mothers during the pregnancy either individually or in combination based on body weight. Though the mothers were taking different drugs with differing combinations, a statistical significance in outcome could not be made out due to low numbers.

The birth weight of babies born to PSS patients was 2.3 ± 0.8 kg. The mean duration of neonatal intensive care unit (NICU) stay was seven days. Fetal echocardiogram revealed complete CHB in six (50%) and incomplete heart block in one baby (8.3%). One baby needed a permanent pacemaker. The mean maternal age in mothers with neonates having a CHB was 29.6 ± 4.8 years. The mean birth weight of babies with CHB was 2.1 ± 0.7 kg. Table 1 summarizes the maternal and fetal outcomes of pregnancy in mothers with PSS, SLE, and RA.

**Table 1:** Maternal and fetal outcomes of pregnancy in mothers with PSS, SLE, and RA CS, cesarean section; IUFD, intrauterine fetal demise; LSCS, lower segment cesarean section; MTP, medical termination of pregnancy; PSS, primary Sjogren’s syndrome; RA, rheumatoid arthritis; SLE, systemic lupus erythematosus; VD, vaginal delivery

Outcome variable	Diagnosis		
	PSS	RA	SLE
	(n = 16)	(n = 2)	(n = 15)
Fetal echo			
Normal	5 (41.7)	2 (100.0)	11 (91.7)
Complete heart block	6 (50.0)	-	1 (8.3)
Intermittent heart block	1 (8.3)	-	-
Mode of delivery			
First trimester MTP	4 (23.5)	-	3 (15.0)
IUFD	2 (11.8)	-	-
Preterm VD	1 (5.9)	1 (50.0)	1 (10.0)
Preterm LSCS	1 (5.9)	-	2 (20.0)
Term VD	3 (17.6)	-	2 (20.0)
Term CS	5 (29.4)	1 (50.0)	7 (35.0)
Mode of delivery			
Normal	6 (50.0)	1 (50.0)	3 (25.0)
LSCS	6 (50.0)	1 (50.0)	9 (75.0)
Birth status			
Preterm	2 (20)	1 (50.0)	3 (25.0)
Term	8 (80)	1 (50.0)	9 (75.0)
Gestational age at delivery	30.9 ± 9.0	36.5 ± 0.7	31.9 ± 11.4
Birth weight	2.3 ± 0.8	2.6 ± 0.04	2.5 ± 0.8

The incidence of heart block, detected with fetal echo, is significantly higher when the mother has PSS compared to other CDs (Table 2). Though the percentage of preterm births was higher in PSS, the values had only borderline significance.

**Table 2:** Comparison of fetal outcomes between PSS and SLE + RE p < 0.05 is considered significant. IUGR, intrauterine growth restriction; PSS, primary Sjogren’s syndrome; RA, rheumatoid arthritis; SLE, systemic lupus erythematosus

Outcome variables	Category	Diagnosis		p-value
		PSS	SLE + RA	
IUGR	0	10 (88.2)	11 (78.5)	0
	1	2 (11.8)	3 (21.4)	
Fetal echocardiogram	Normal	5 (41.7)	13 (92.8)	0.004
	Heart block	7 (58.3)	1 (7.2)	
Need for intervention	No	7 (87.5)	-	NA
	Yes	1 (12.5)	-	
Permanent pacemaker	No	11 (91.6)	13 (92.8)	0.9
	Yes	1 (8.3)	1 (7.2)	
Birth status	Preterm	4 (33.3)	4 (28.6)	0.06
	Term	8 (66.7)	10 (71.4)	
Birth weight		2.3 ± 0.8	2.5 ± 0.8	0.372

## Discussion

Researchers acknowledge that women are more prone than men to be diagnosed with autoimmune disorders. Autoimmune illness in pregnancy has been linked to an increased likelihood of complications for both mother and fetus, requiring interdisciplinary care and vigilant monitoring [15]. Numerous studies have shown the highly variable rate of fetal and maternal risks in patients with SSJ [7-12]. In India, there is a paucity of data on the incidence and prevalence of these outcomes in pregnant women with PSS. Here, we report a retrospective study on maternal and fetal outcomes of pregnant women with PSS.

Based on the literature that is currently available, there is a significant risk of preeclampsia, PPROM, cesarean birth, pulmonary embolism/DVT, and prolonged hospital stays for pregnant patients with SSJ. It is seen that prematurity is frequent among babies born to SSJ patients, with associated IUGR and congenital malformations [16]. Based on the American-European Consensus Criteria, the prevalence of PSS in the general female population has been estimated to range between 0.1% and 4.8% [17]. Elliott et al., however, have shown a frequency of 0.013 [16]. Selection bias and misclassification have been blamed for the large range of prevalence estimates. The incidence of PSS in our study was 1/1,000 pregnancies per year. The prevalence of pregnancies with SSJ has increased over the last few years, possibly due to increased awareness and better diagnostic techniques [4].

Patients with PSS should undergo a thorough evaluation for disease activity and potential risks to the health of the mother and fetus before considering pregnancy. The disease activity rating system known as the EULAR Sjogren's syndrome disease activity index (ESSDAI) has recently been introduced. It encompasses the glandular, constitutional, lymphadenopathic, cutaneous, respiratory, renal, articular, muscular, peripheral and central neurological, and biological domains [5]. It is advised that a minimum of six months prior to pregnancy, PSS disease activity should be under adequate control [18]. During the periconceptional stage, teratogenic medications such as methotrexate, cyclophosphamide, and mycophenolate should be stopped. To reduce the risk of illness flares, rheumatology follow-up and regular monitoring are advised when switching to pregnancy-safe medications. During pregnancy, prednisolone, azathioprine, cyclosporine, and tacrolimus can be used to manage illness. Steroid use can lead to small-for-gestational-age babies, diabetes, hypertension, preeclampsia, oligohydramnios, premature membrane rupture, adrenal insufficiency, and neurological abnormalities in the progeny. As such, it is recommended to use alternative immunosuppressive medications and lesser dosages of steroids [19].

The high incidence of preterm deliveries in our series (33%) compares with other studies that report consistently high preterm deliveries [20]. Most studies on women with CD show a significantly increased relative risk of preeclampsia, premature delivery, cesarean birth, and IUGR [21]. Patients with well-controlled disease activity, as measured using the ESSDAI scoring system, show promising outcomes compared with those that are not well controlled [22]. However, in our cohort, there was only a borderline significant increase in preterm delivery (p = 0.06).

Neonatal lupus and CHBs are the serious fetal complications of autoimmune pregnancies associated with the presence of anti-Ro/SSA and anti-La/anti-Sjogren's-syndrome-related antigen B (SSB) antibodies [23]. Every second neonate in our study had CHB. Demarchi et al. reported one incidence of CHB in a series of 18 pregnancies with PSS. The incidence in literature for CHB among neonates born to mothers with PSS is 2% [24]. The high incidence in our series may be due to inadequate early diagnosis and poor control of disease activity. Further, the low prevalence and lack of awareness of complications for SSJ in Indian patients prevent physicians from diagnosing the disease early and controlling the severity of disease activity before planning pregnancy.

One baby in our series required a pacemaker. CHB carries a high morbidity and mortality rate in most babies [23]. In many studies, treating intrauterine fetal atrioventricular block is often disappointing, and more than 90% of affected newborns undergo definitive pacemaker implantation [25]. Cardiac involvement can manifest at delivery or in the early postnatal period, but it typically develops in utero between 16 and 24 weeks [26]. For fetal screening, there are no firm evidence-based recommendations. The majority of obstetricians adhere to the weekly fetal echocardiography monitoring regimen during this time, given the risk of developing cardiac events is highest between 16 and 28 weeks [24].

There is no suggested effective treatment to stop the significant cardiac injury from getting worse. Fluorinated steroids (Dexamethasone 4-9 mg/day for 3-19 weeks or Betamethasone 12-24 mg/week) have been widely used. Further, concerns have been raised regarding the maternal and fetal adverse effects of steroids [19]. Treatment options other than steroids are intravenous immunoglobulins (IVIG) at 400 mg/kg [27]. Plasmapheresis and adrenergic receptor agonists showed some benefit in halting the degree of heart block progression [28,29]. It has been observed that hydroxychloroquine used during pregnancy protects against cutaneous and cardiac symptoms [30].

Limitations

This is a single-center retrospective study of a rare autoimmune disorder with an incidence of <1/1,000 pregnancies/year and its effect on feto-maternal outcomes. Hence, the numbers are too small to project its effect on the country's overall data. Many neonates and children with CHB, born to mothers with PSS, must have directly visited the pediatric cardiology and may not have been registered in our study. A more detailed multicentric ambispective study, including departments of gynecology, cardiology, rheumatology, perinatology, and neonatology, will yield more data. Since many women change hospitals for second opinions and further follow-up, the data of such patients may then be lost. We are working on extending the scope of this study to include all patients with Sjogren’s and also compare effects on first and subsequent pregnancies vs other autoimmune disorders like SLE, RA, etc. The effects of first versus second pregnancy in our series could not be studied due to small numbers.

## Conclusions

Pregnancy in patients with PSS is more common than thought in India. Babies born to SSJ patients are likely premature and have IUGR and congenital malformations. Maternal and fetal complications are high in this set of patients as compared to other connective tissue disorders. Every second neonate in our study had CHB. Poor control of disease activity, low prevalence, and lack of awareness of complications for SSJ in Indian patients prevent physicians from diagnosing the disease early and controlling the severity of disease activity before planning pregnancy. Early detection, pre-conceptional counseling, and proper workup by a multidisciplinary approach may improve the outcome in such patients.
